# Development and external validation of an admission-based model for 180-day mortality in diabetic acute myocardial infarction

**DOI:** 10.3389/fendo.2026.1891663

**Published:** 2026-07-02

**Authors:** Yanlong Zhao, Haodong Jiang, Yuanyuan Zhao, Shuai Wang, Qicheng Yu, Jing Zeng, Shan Xie, Jiatong Li, Zhi Liu

**Affiliations:** 1 Department of Emergency, Xuanwu Hospital, Capital Medical University, Beijing, China; 2 Department of Medical Care, Beijing Luhe Hospital, Capital Medical University, Beijing, China

**Keywords:** acute myocardial infarction, diabetes mellitus, external validation, mortality prediction, prognostic model, risk stratification

## Abstract

**Background:**

Patients with diabetes mellitus and acute myocardial infarction (DM-AMI) are at high risk, but risk heterogeneity within this subgroup remains insufficiently characterized. Whether a parsimonious admission-based model provides prognostic information beyond GRACE remains uncertain. We aimed to assess admission risk heterogeneity in DM-AMI and develop and externally validate a model for 180-day mortality using routine variables.

**Methods:**

This retrospective dual-cohort study included AMI patients from a Beijing tertiary hospital (development cohort) and an ICU-based MIMIC-IV cohort (external validation). A Cox model was built using admission variables. Model performance was evaluated using discrimination, calibration, and decision curve analysis with internal bootstrap validation and comparison with GRACE.

**Results:**

Among 4,167 patients, 1,514 had DM-AMI with 90 deaths at 180 days. The model included eight variables (age, heart rate, SBP, glucose, BUN, hemoglobin, RDW, WBC). It showed good discrimination in the development cohort (C-index 0.848; AUC 0.857) and maintained performance externally (AUC 0.744). GRACE comparison showed similar discrimination, while the combined model improved performance (ΔAUC = 0.014, P = 0.031). Calibration in MIMIC-IV indicated risk overestimation (slope 0.527; O/E 0.652).

**Conclusion:**

DM-AMI patients exhibit marked admission risk heterogeneity. The model provides complementary prognostic information beyond GRACE but requires recalibration for absolute risk use in external settings.

## 1 Introduction

Acute myocardial infarction (AMI) remains one of the leading causes of death and disability worldwide (1, 2). With the rising prevalence of diabetes mellitus, an increasing proportion of patients presenting with AMI also have concomitant diabetes, which is widely recognized as an adverse prognostic factor in this setting (3, 4). Previous studies have shown that, compared with patients without diabetes, those with diabetes-associated AMI generally have a greater burden of comorbidities, more complex clinical management, and a higher risk of both short- and long-term mortality (5, 6). Accordingly, AMI in the context of diabetes mellitus has become a clinically important high-risk subgroup.

However, existing evidence has mainly emphasized the overall adverse effect of diabetes on AMI prognosis, whereas relatively limited attention has been paid to heterogeneity of risk within the population of patients with diabetes-associated AMI (DM-AMI) (7). In this context, risk heterogeneity refers to variations in admission clinical, hemodynamic, metabolic, renal, inflammatory, and hematologic profiles that may correspond to substantially different mortality risks within the same diagnostic subgroup (8, 9). Although the Global Registry of Acute Coronary Events (GRACE) score is widely used for risk stratification in patients with acute coronary syndromes, whether routinely available admission clinical and laboratory variables can provide prognostic information complementary to GRACE in patients with DM-AMI remains uncertain (10–13). Moreover, despite the availability of several prognostic models for AMI, relatively few studies have specifically focused on patients with DM-AMI or evaluated intermediate-term mortality risk using routinely available admission data. Therefore, whether clinically meaningful risk heterogeneity already exists at hospital admission in patients with DM-AMI, and whether such heterogeneity can be quantified using routine information, remains insufficiently addressed.

Recent advances in digital-health and risk-stratified management strategies have highlighted the importance of early identification of high-risk patients with diabetes mellitus (14). Against this background, we aimed to evaluate admission risk heterogeneity among patients with DM-AMI in a real-world hospitalized AMI cohort and, on this basis, to develop a parsimonious prediction model for 180-day all-cause mortality using routinely available early clinical and laboratory variables. We further compared its performance with that of the GRACE score, assessed its incremental prognostic value beyond GRACE, and externally validated the model in an ICU-based MIMIC-IV cohort to evaluate both discrimination and calibration transportability.

## 2 Methods

### 2.1 Data sources

This was a retrospective, dual-cohort observational study designed to assess admission risk heterogeneity in patients with DM-AMI, to develop a prediction model for 180-day all-cause mortality based on routinely available early clinical and laboratory data, and to examine its transportability in an independent external cohort. The development cohort was derived from consecutive adult patients hospitalized at a tertiary hospital in Beijing between January 2016 and July 2024 with a confirmed diagnosis of AMI. Demographic characteristics, comorbidities, vital signs, laboratory data, and follow-up information were extracted from the electronic medical record system. The study protocol was approved by the local institutional ethics committee, with a waiver of informed consent.

The external validation cohort was obtained from the MIMIC-IV version 3.0 database, which contains de-identified clinical data from patients admitted to the intensive care units of Beth Israel Deaconess Medical Center, United States, between 2008 and 2022 (15, 16). The database includes baseline characteristics, diagnostic codes, laboratory results, medication records, procedures, and outcome data, and mainly reflects the profile of critically ill patients managed in intensive care settings. Access to the database was obtained after completion of the required PhysioNet credentialing procedures, including CITI training (“Data or Specimens Only Research”; certification number: 63998837) and acceptance of the data use agreement. Because MIMIC-IV contains fully de-identified data, additional informed consent was not required.

### 2.2 Study population and cohort assembly

In the development cohort, we included adult patients aged 18 years or older who were consecutively hospitalized with a confirmed diagnosis of AMI. AMI was defined according to the Fourth Universal Definition of Myocardial Infarction and included both ST-segment elevation myocardial infarction and non-ST-segment elevation myocardial infarction (17). For patients with multiple hospitalizations, only the first eligible admission was retained as the index admission.

Within the overall AMI population, diabetes mellitus was identified from the electronic medical record system based on documented medical history and structured clinical diagnosis records during the index hospitalization, and patients meeting these criteria were classified as the DM-AMI study population. All baseline variables were defined using the first structured measurements recorded within 24 hours after admission. Only patients with available 180-day outcome data were included in the analysis. Candidate predictors with missingness below the prespecified threshold were handled by imputation before model development. Exclusion criteria were age <18 years, missing 180-day follow-up data, and duplicate admissions. In addition, patients without diabetes mellitus and those lacking variables required for model development were excluded from the final DM-AMI modeling cohort.

**Figure 1:**
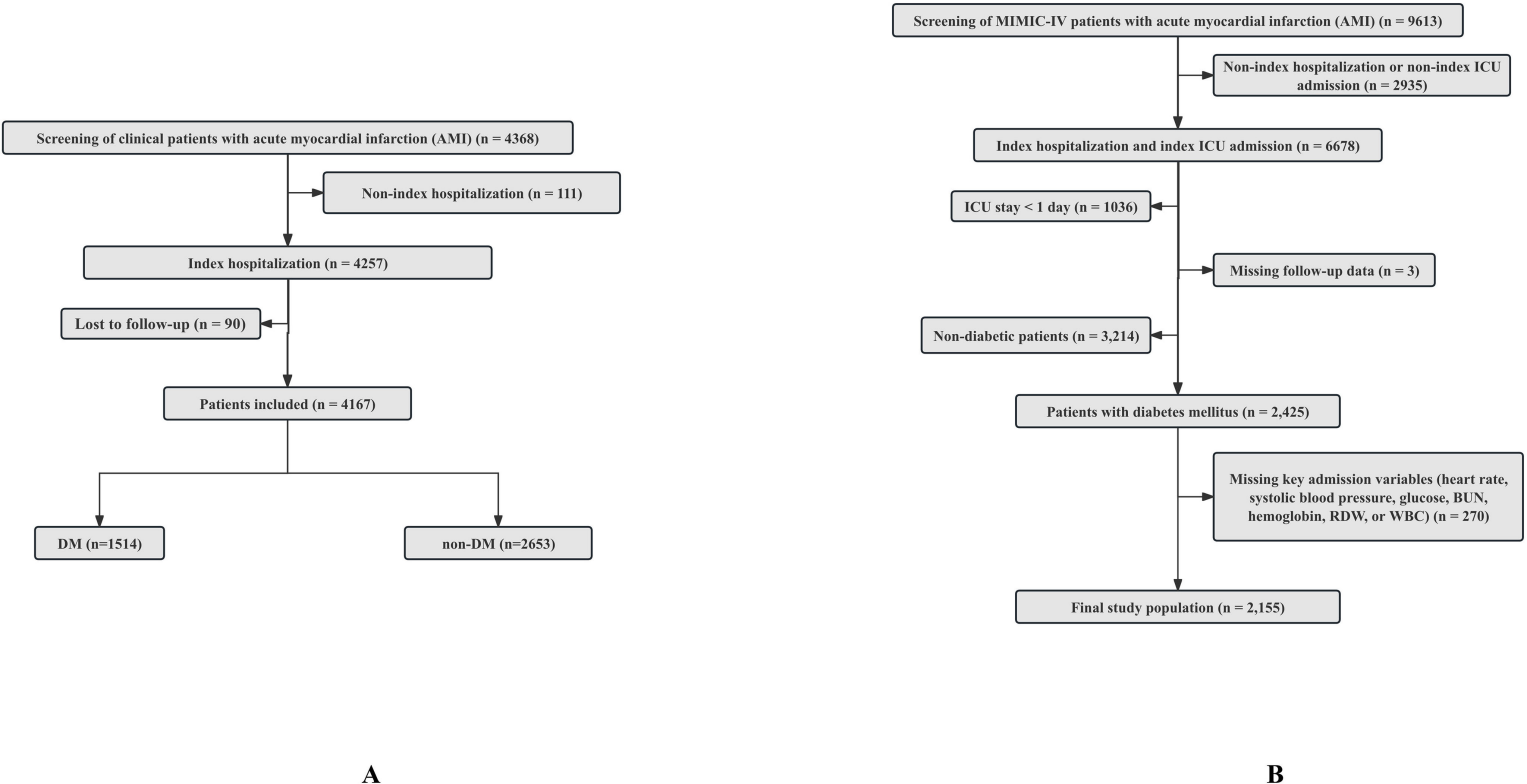
Study flowchart of the development and external validation cohorts. Flowchart of patient selection in the development **(A)** and external validation **(B)** cohorts. A total of 1,514 patients with diabetes mellitus were included in the development cohort. In the MIMIC-IV cohort, 2,155 patients with diabetes mellitus were included after applying predefined inclusion and exclusion criteria. The exclusion for missing key admission variables refers to patients whose required validation information could not be ascertained before model application; sporadic low-level baseline missingness among retained patients was handled during preprocessing as described in the Methods.

For external validation, patients with AMI in MIMIC-IV were identified from structured ICD-9 and ICD-10 diagnosis records, and the first ICU admission was defined as the index admission. Diabetes mellitus in MIMIC-IV was identified from hospital-admission-level diagnosis records using diabetes-related ICD-9 and ICD-10 codes, corresponding to ICD-9 code 250.xx and ICD-10 codes E10–E14. Candidate predictor selection was performed exclusively in the development cohort. The external cohort was used only to evaluate the performance of the prespecified final model and did not contribute to variable selection. Variables retained in the final model were mapped to the external dataset, and patients aged 18 years or older with diabetes mellitus, available model variables, and ascertainable 180-day outcomes were included in the validation analysis. Differences in variable availability between the two cohorts were handled pragmatically in a manner consistent with external validation, without altering the original model structure. The patient selection process is shown in **Figure 1**.

### 2.3 Variables and definitions

In the development cohort, baseline variables were extracted from the first structured records obtained within 24 hours of admission, including demographic characteristics, vital signs, laboratory tests, comorbidities, cardiac functional status, and treatment-related information. Demographic variables included age and sex. Vital signs included heart rate (HR), systolic blood pressure (SBP), and diastolic blood pressure. Laboratory variables included glucose, glycated hemoglobin (HbA1c), creatinine (Cr), blood urea nitrogen (BUN), hemoglobin (Hb), white blood cell count (WBC), red cell distribution width (RDW), platelet count, albumin, prealbumin, high-sensitivity C-reactive protein (hs-CRP), interleukin-6 (IL-6), and D-dimer. Clinical variables included STEMI, Killip class, chronic kidney disease (CKD), atrial fibrillation (AF), chronic obstructive pulmonary disease (COPD), smoking status, percutaneous coronary intervention (PCI), culprit vessel, and left ventricular ejection fraction (LVEF). Admission GRACE and CRUSADE scores were also extracted. Diabetes status was identified from prior diagnoses or structured variables in the medical record.

Candidate predictors were prespecified on the basis of clinical relevance, early availability at admission, data completeness, interpretability, and potential association with outcome. Predictor retention was not based on univariable statistical significance alone. Variables were evaluated using multivariable associations, clinical plausibility, stability, collinearity, missingness, cross-cohort availability, and model parsimony. Collinearity was assessed using variance inflation factors (VIFs). To improve generalizability, we prioritized routinely available variables that could be more easily standardized across centers. The external validation cohort did not participate in predictor selection. GRACE was treated as a comparator rather than as a candidate component of the new model because it overlaps conceptually and mathematically with several admission variables, including age, heart rate, systolic blood pressure, renal function, and disease-severity indicators.

In the external validation cohort, variables retained in the final model were extracted and mapped to the corresponding MIMIC-IV variables. The core variables used for validation were age, HR, SBP, glucose, BUN, Hb, RDW, and WBC. Laboratory values with different units were converted to common units as follows: glucose from mg/dL to mmol/L, BUN from mg/dL to mmol/L, Hb from g/dL to g/L, and creatinine from mg/dL to μmol/L. Patients missing any of the eight prespecified model predictors or 180-day outcome/follow-up information were excluded before model application. Sporadic missingness in non-model baseline covariates used for descriptive summaries was handled using the non-parametric random-forest missForest algorithm when required; outcome status and follow-up time were not imputed.

### 2.4 Outcome definition

The primary outcome was 180-day all-cause mortality after the index admission. In the development cohort, survival status was determined by integrating information from the electronic medical record system and structured follow-up data. In the MIMIC-IV cohort, 180-day all-cause mortality was calculated from the index ICU admission, and deaths occurring after hospital discharge but within 180 days were also included. For time-to-event analyses, follow-up time was defined as the interval from the index admission to death or censoring. Patients who survived beyond 180 days were censored at 180 days, whereas patients who died within 180 days were treated as having experienced the event. Outcome status and follow-up time were not imputed.

### 2.5 Risk heterogeneity assessment and model development

In the development cohort, we assessed admission risk heterogeneity among patients with DM-AMI using the GRACE score. Patients were categorized into low-risk (≤140), intermediate-risk (141–170), and high-risk (>170) groups according to predefined cutoffs. The threshold of >140 corresponds to guideline-recognized high risk in acute coronary syndromes, whereas the cutoff of 170 was used to further characterize a very-high-risk subgroup (8, 9). Kaplan–Meier methods were used to estimate 180-day survival probabilities across groups, and differences were compared using the log-rank test.

Subsequently, a prediction model for 180-day all-cause mortality was developed in the development cohort. Admission candidate variables were first evaluated in univariable Cox proportional hazards analyses, after which prespecified candidate predictors were entered into a multivariable Cox proportional hazards model. Final variable retention was determined not by statistical significance alone but by considering clinical interpretability, predictor stability, collinearity, missingness, cross-cohort availability, and model parsimony. The final model retained eight variables: age, heart rate, systolic blood pressure, glucose, blood urea nitrogen, hemoglobin, red cell distribution width, and white blood cell count. All continuous predictors were entered as continuous variables. The proportional hazards assumption was assessed using Schoenfeld residuals.

To further characterize dose–response relationships between continuous predictors and mortality, restricted cubic spline (RCS) analyses were performed for glucose, BUN, hemoglobin, and RDW, with tests for both overall and nonlinear effects. Because BUN and hemoglobin showed evidence of nonlinearity, sensitivity analyses incorporating RCS terms were performed, whereas the primary model retained linear terms to preserve parsimony and interpretability. Based on the final multivariable Cox model, an individual linear predictor (lp_main) was calculated for each patient. A model-driven risk stratification framework was then constructed using tertiles of lp_main, categorizing patients into low-, intermediate-, and high-risk groups. Kaplan–Meier curves were used to estimate 180-day survival across these groups, and differences were compared using the log-rank test.

### 2.6 Model performance and validation

In the development cohort, model discrimination and overall predictive accuracy were assessed using Harrell’s C-index, the area under the receiver operating characteristic curve (AUC) at 180 days, and the Brier score at 180 days. Individual predicted risks of 180-day mortality were derived from the estimated 180-day baseline survival function of the Cox model combined with each patient’s linear predictor. Calibration was assessed by grouped calibration analysis and calibration plots. Internal validation was performed using bootstrap resampling with 1,000 repetitions, and optimism-corrected C-index and calibration slope were calculated. The observed-to-expected (O/E) event ratio was additionally calculated to assess calibration performance.

For external validation, the final model developed in the derivation cohort was directly applied to the MIMIC-IV cohort. Model performance was evaluated using the 180-day AUC, Brier score, calibration slope, calibration intercept, O/E ratio, and grouped calibration. To compare prognostic performance, the final model was evaluated against the GRACE score using DeLong tests, and a combined model incorporating both GRACE and the final model linear predictor was also assessed. Decision curve analysis was performed to compare potential clinical utility.

### 2.7 Statistical analysis

All statistical analyses were performed using R software, version 4.5.2. Continuous variables were summarized as mean ± standard deviation for normally distributed data or as median with interquartile range (IQR) for non-normally distributed data, whereas categorical variables were summarized as frequencies and percentages. Group comparisons were performed using t tests, Wilcoxon rank-sum tests, chi-square tests, or Fisher’s exact tests, as appropriate. Missingness was assessed before model development. Variables with a missing proportion of ≥20% were excluded from the candidate predictor set, whereas variables with a missing proportion of <20% were imputed using the missForest algorithm (18). Patients with missing 180-day outcome data were excluded from analysis. All tests were two-sided, and a *P* value <0.05 was considered statistically significant.

## 3 Results

### 3.1 Study population and baseline characteristics

A total of 4,167 patients with AMI were included in the clinical development cohort, of whom 1,514 had diabetes mellitus and were classified as the DM-AMI population, whereas 2,653 did not have diabetes mellitus. Among the 1,514 patients with DM-AMI, 90 deaths occurred within 180 days, whereas 1,424 survived. Baseline characteristics of the development cohort are presented in **Table 1**. Compared with patients without diabetes, those with DM-AMI exhibited a heavier burden of abnormalities at admission. Specifically, the DM-AMI group was older, had a higher admission heart rate, and showed higher levels of glucose and HbA1c. They also had higher blood urea nitrogen levels and lower albumin and prealbumin levels. In addition, patients with DM-AMI showed higher levels of inflammatory and thrombosis-related markers, including hs-CRP, IL-6, and D-dimer, as well as higher GRACE and CRUSADE risk scores. These findings suggest that patients with DM-AMI already present with a more complex profile of metabolic disturbance, inflammation, nutritional impairment, and overall risk at an early stage of hospitalization.

**Table 1:** Baseline characteristics of patients with AMI according to diabetes status.

Characteristic	Total (n=4167)	non-DM (n=2653)	DM (n=1514)	*P*-value
Age, years	63.00(55.00,71.00)	62.00(54.00,70.00)	64.00(57.00,72.00)	<0.0001
Sex, n (%)				<0.0001
Female	952(22.85)	517(19.49)	435(28.73)	
Male	3215(77.15)	2136(80.51)	1079(71.27)	
HR, bpm	76.00(68.00,86.00)	75.00(66.00,84.00)	78.00(70.00,88.00)	<0.0001
SBP, mmHg	129.00(116.00,144.00)	129.00(116.00,144.00)	130.00(117.00,144.00)	0.05
DBP, mmHg	75.00(66.00,85.00)	75.00(67.00,85.00)	74.00(66.00,83.00)	<0.0001
GLU, mmol/L	6.23(5.26,8.16)	5.71(5.05,6.56)	8.28(6.24,11.61)	<0.0001
HbA1c, %	6.10(5.60,7.00)	5.76(5.50,6.20)	7.38(6.30,8.68)	<0.0001
Cr, µmol/L	73.00(63.00,89.70)	73.00(63.00,87.00)	73.00(62.00,89.70)	0.13
BUN, mmol/L	5.80(4.62,7.04)	5.57(4.47,6.69)	6.35(4.91,7.68)	<0.0001
Hb, g/L	138.00(128.00,149.00)	140.00(130.00,150.00)	137.13(124.00,145.00)	<0.0001
WBC, ×10^9^/L	9.17(7.42,11.06)	9.24(7.44,11.23)	9.10(7.37,10.72)	0.01
RDW	12.90(12.40,13.20)	12.90(12.50,13.30)	12.90(12.40,13.20)	0.11
GRACE Score	150.00(128.00,175.00)	147.00(125.00,172.00)	155.00(134.00,180.00)	<0.0001
CRUSADE Score	22.00(13.00,36.00)	19.00(10.00,30.00)	30.00(19.00,43.00)	<0.0001
LVEF, %	59.00(51.00,63.00)	59.00(52.00,63.00)	58.00(50.00,62.23)	<0.0001
STEMI, n (%)				<0.0001
No	1482(35.57)	863(32.53)	619(40.89)	
Yes	2685(64.43)	1790(67.47)	895(59.11)	
AF, n (%)				0.79
No	4013(96.30)	2557(96.38)	1456(96.17)	
Yes	154(3.70)	96(3.62)	58(3.83)	
CKD, n (%)				<0.0001
No	3809(91.41)	2482(93.55)	1327(87.65)	
Yes	358(8.59)	171(6.45)	187(12.35)	
Killip class, n (%)				<0.0001
1	2658(63.79)	1794(67.62)	864(57.07)	
2	1092(26.21)	631(23.78)	461(30.45)	
3	312(7.49)	179(6.75)	133(8.78)	
4	105(2.52)	49(1.85)	56(3.70)	
PCI, n (%)				<0.001
No	1528(36.67)	918(34.60)	610(40.29)	
Yes	2639(63.33)	1735(65.40)	904(59.71)	

Baseline Characteristics of Patients With AMI According to Diabetes Status. Data are presented as median (IQR) or n (%). P values were calculated using the Wilcoxon rank-sum test and χ² test (or Fisher exact test, as appropriate). This concise main table retains core demographic, hemodynamic, laboratory, risk-score, cardiac-function, and treatment variables. The extended baseline characteristics are provided in **Supplementary Table 9**. AMI, acute myocardial infarction; DM, diabetes mellitus; HR, heart rate; SBP, systolic blood pressure; DBP, diastolic blood pressure; GLU, glucose; HbA1c, glycated hemoglobin; Cr, creatinine; BUN, blood urea nitrogen; Hb, hemoglobin; WBC, white blood cell count; RDW, red cell distribution width; GRACE, Global Registry of Acute Coronary Events score; CRUSADE, Can Rapid risk stratification of Unstable angina patients Suppress ADverse outcomes with Early implementation of the ACC/AHA guidelines score; LVEF, left ventricular ejection fraction; STEMI, ST-segment elevation myocardial infarction; AF, atrial fibrillation; CKD, chronic kidney disease; PCI, percutaneous coronary intervention.

### 3.2 Initial evidence of within-group risk heterogeneity in diabetic AMI

**Figure 2:**
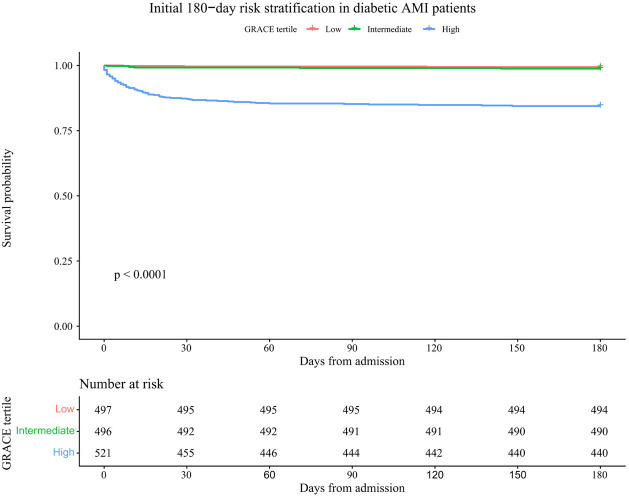
Kaplan-Meier curves for 180-day survival according to GRACE-based initial risk stratification in patients with diabetic AMI. Kaplan-Meier curves for 180-day survival according to GRACE-based initial risk stratification in patients with diabetic AMI. Patients were classified using admission GRACE score cutoffs of 140 and 170. The threshold of >140 corresponds to guideline-recognized high risk in ACS and is supported by the original GRACE risk scale (10), and >170 was used to further identify a very-high-risk stratum.

To explore risk heterogeneity within the DM-AMI population, we first performed stratification according to the admission GRACE score. Using predefined GRACE score cutoffs of ≤140, 141–170, and >170, patients were classified into low-risk (n = 497), intermediate-risk (n = 496), and high-risk (n = 521) groups. The numbers of deaths and 180-day mortality rates across these groups are shown in **Table 2**. Marked differences in 180-day mortality were observed among the three strata, with mortality rates of 0.60%, 1.21%, and 15.55% in the low-, intermediate-, and high-risk groups, respectively. Kaplan–Meier survival curves demonstrated clear separation across the three groups. Patients in the high-risk group showed substantially worse survival from the early phase of follow-up and remained consistently below the other groups throughout the 180-day period, whereas some separation was also observed between the low- and intermediate-risk groups. The between-group difference was statistically significant (log-rank P < 0.0001; **Figure 2**). These findings provided initial evidence that clinically relevant risk heterogeneity was already present at admission among patients with DM-AMI.

**Table 2:** 180-day mortality according to GRACE score–based risk stratification in patients with diabetic AMI.

Risk group (GRACE score).	Patients, n	Deaths, n	Mortality, %
Low (≤140)	497	3	0.60%
Intermediate (141–170)	496	6	1.21%
High (>170)	521	81	15.55%

180-Day Mortality According to GRACE Score-Based Risk Stratification in Patients with Diabetic AMI. Data are presented as n or %. Patients were grouped by admission GRACE score (<=140, 141-170, and >170). The threshold of >140 corresponds to guideline-recognized high risk in ACS and is supported by the original GRACE risk scale (10), whereas >170 was used to further describe a very-high-risk stratum. AMI, acute myocardial infarction; GRACE, Global Registry of Acute Coronary Events.

### 3.3 Identification of admission predictors and model construction

To further identify factors associated with risk heterogeneity in DM-AMI, we evaluated routinely available admission clinical and laboratory variables using Cox proportional hazards regression. In univariable analyses, older age, higher heart rate, higher glucose, creatinine, BUN, RDW, and WBC, as well as higher hs-CRP, IL-6, and D-dimer levels, were associated with increased risk of 180-day mortality. In contrast, higher SBP, hemoglobin, albumin, and baseline LVEF were associated with lower mortality risk. HbA1c was not significantly associated with the outcome, whereas STEMI showed only borderline significance (**Supplementary Table 1**).

These candidate variables were subsequently entered into multivariable Cox regression analysis. In the multivariable model, age, heart rate, glucose, BUN, RDW, and WBC remained independently associated with increased 180-day mortality, whereas SBP and hemoglobin were inversely associated with risk. Sex, STEMI, creatinine, and albumin did not retain independent statistical significance (**Supplementary Table 2**). On the basis of these findings, we constructed a final parsimonious model comprising eight core admission variables: age, heart rate, SBP, glucose, blood urea nitrogen, hemoglobin, red cell distribution width, and white blood cell count. The corresponding hazard ratios were 1.041, 1.018, 0.979, 1.064, 1.056, 0.982, 1.234, and 1.100, respectively (**Table 3**). Collinearity among the final predictors was low, with variance inflation factors ranging from 1.04 to 1.34. Testing of the proportional hazard assumption indicated that the final Cox model satisfied the assumption overall (global P = 0.1046).

**Table 3:** Final multivariable Cox model for 180-day mortality in patients with diabetic AMI.

Variable	HR (95% CI)	*P value*
Age	1.041 (1.021–1.061)	<0.001
Heart rate	1.018 (1.008–1.028)	<0.001
SBP	0.979 (0.968–0.990)	<0.001
Glucose	1.064 (1.025–1.104)	0.001
BUN	1.056 (1.027–1.087)	<0.001
Hemoglobin	0.982 (0.971–0.993)	0.001
RDW	1.234 (1.025–1.485)	0.026
WBC	1.100 (1.042–1.162)	<0.001

Final Multivariable Cox Model for 180-Day Mortality in Patients with Diabetic AMI. Data are presented as hazard ratios (HR) with 95% confidence intervals (CI). HRs were derived from multivariable Cox proportional hazards regression analysis. AMI, acute myocardial infarction; SBP, systolic blood pressure; BUN, blood urea nitrogen; RDW, red cell distribution width; WBC, white blood cell count.

**Figure 3:**
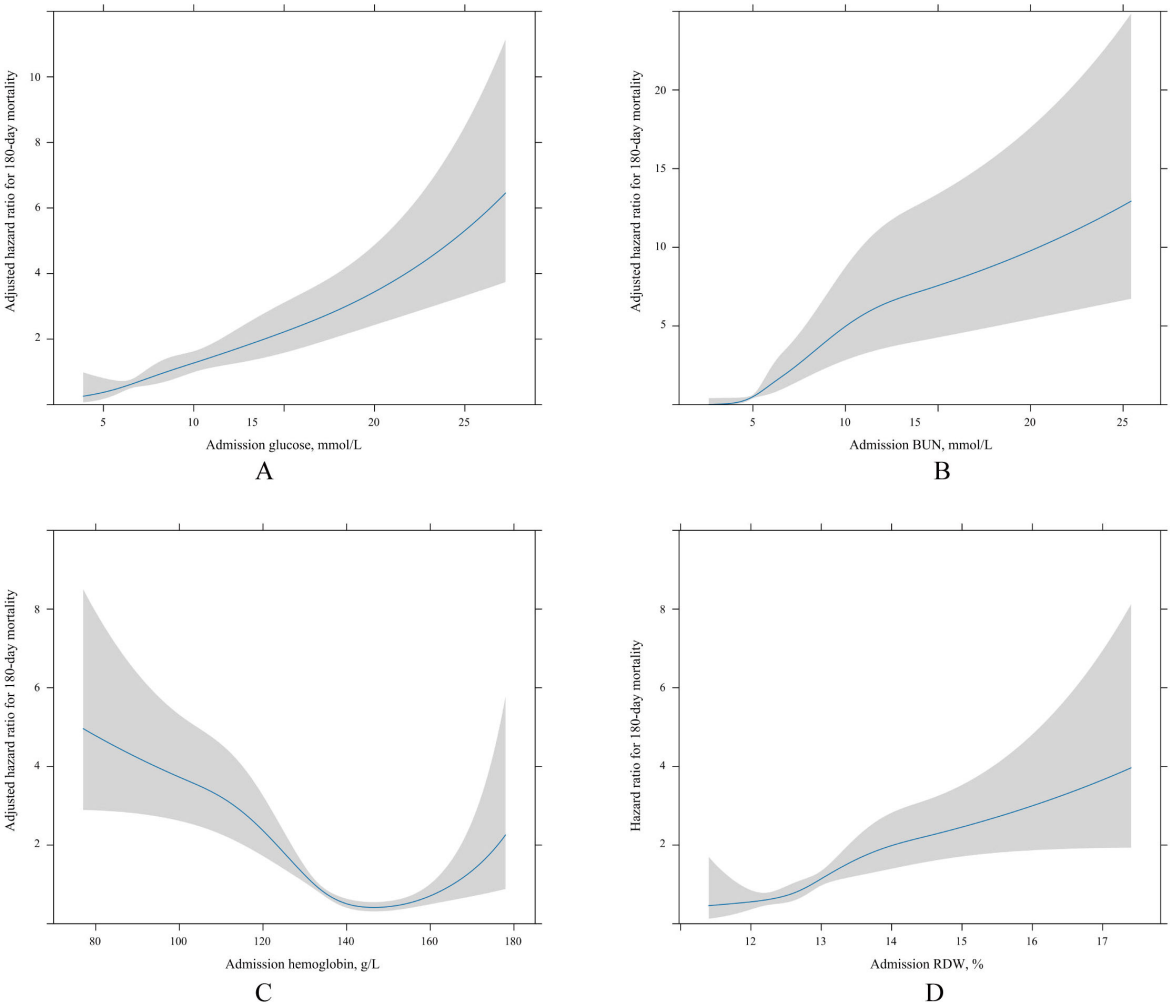
Restricted cubic spline analysis of the associations between admission biomarkers and 180-day mortality in patients with diabetic AMI. Restricted cubic spline analysis of the associations between admission biomarkers and 180-day mortality in patients with diabetic AMI. Panels show the adjusted hazard ratios for 180-day mortality according to admission glucose **(A)**, BUN **(B)**, hemoglobin **(C)**, and RDW **(D)**. Solid lines represent estimated hazard ratios, and shaded areas indicate 95% confidence intervals. Glucose showed an approximately linear association with risk, whereas BUN and hemoglobin showed nonlinear associations; RDW showed a significant overall association without clear evidence of nonlinearity.

Restricted cubic spline analyses further showed that glucose was significantly associated with 180-day mortality in an approximately linear manner. In contrast, BUN and hemoglobin displayed nonlinear associations with mortality risk, whereas RDW showed a significant overall association without clear evidence of nonlinearity (**Figures 3A–D**; **Supplementary Table 3**). Sensitivity analyses incorporating spline terms for BUN and hemoglobin improved apparent model fit and discrimination; however, the primary linear model was retained to preserve parsimony and interpretability. These findings suggest that admission predictors may capture outcome risk through different dose–response patterns rather than through a single common threshold effect.

### 3.4 Final risk stratification framework in the development cohort

We next constructed a model-driven risk stratification framework based on the linear predictor generated from the final model (lp_main). Using tertiles of lp_main, with cutoffs at −0.474014 and 0.428336, patients with DM-AMI were classified into low-risk (n = 505), intermediate-risk (n = 504), and high-risk (n = 505) groups, yielding a well-balanced distribution of sample size across strata.

**Figure 4:**
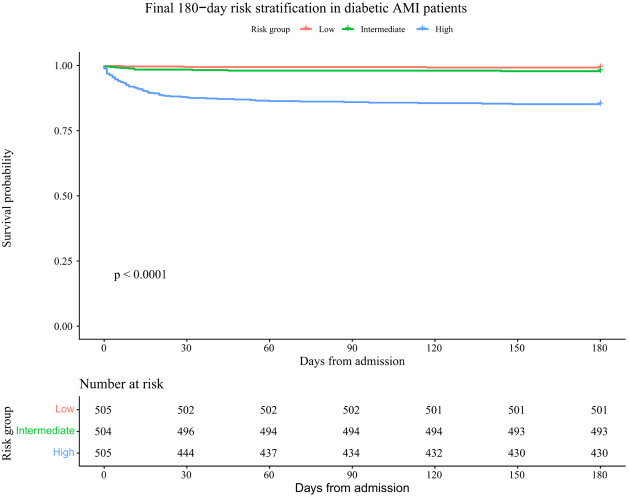
Kaplan-Meier curves for 180-day survival according to final model-based risk stratification in patients with diabetic AMI. Kaplan-Meier curves for 180-day survival according to final model-based risk stratification in patients with diabetic AMI. Risk groups were defined according to tertiles of the linear predictor derived from the final multivariable model. The corresponding group sizes were 505, 504, and 505 for the low-, intermediate-, and high-risk groups, respectively. Survival differed significantly across groups (log-rank P < 0.0001).

Kaplan–Meier curves showed clear separation in 180-day survival among the three groups. The high-risk group exhibited a survival disadvantage from the early phase of follow-up and remained consistently below the other groups throughout the 180-day period. A modest but visible separation was also observed between the low- and intermediate-risk groups. The overall difference among groups was statistically significant (log-rank P < 0.0001; **Figure 4**), indicating that the final model was able to translate admission information into clinically meaningful risk stratification within the DM-AMI population.

### 3.5 Internal validation in the development cohort

We further evaluated model performance in the development cohort in terms of discrimination, prediction error, and calibration. The final eight-variable model showed good apparent discrimination, with a C-index of 0.848 and a 180-day AUC of 0.857. The Brier score was 0.045, and the observed-to-expected (O/E) event ratio was 1.023 (**Table 4**).

**Table 4:** Performance of the final eight-variable model for predicting 180-day mortality in the development cohort.

Metric	Estimate (95% CI)
C-index	0.848 (0.809-0.887)
AUC at 180 days	0.857 (0.817-0.897)
Brier score at 180 days	0.045
Observed/expected event ratio	1.023
Bootstrap repetitions	1,000
Optimism-corrected C-index	0.838
Optimism-corrected calibration slope	0.947

Performance of the Final Eight-Variable Model for Predicting 180-Day Mortality in the Development Cohort. Data are presented as estimates with 95% confidence intervals (CI), where applicable. Internal validation was performed using 1,000 bootstrap resamples. AUC, area under the receiver operating characteristic curve; O/E, observed-to-expected event ratio.

**Figure 5:**
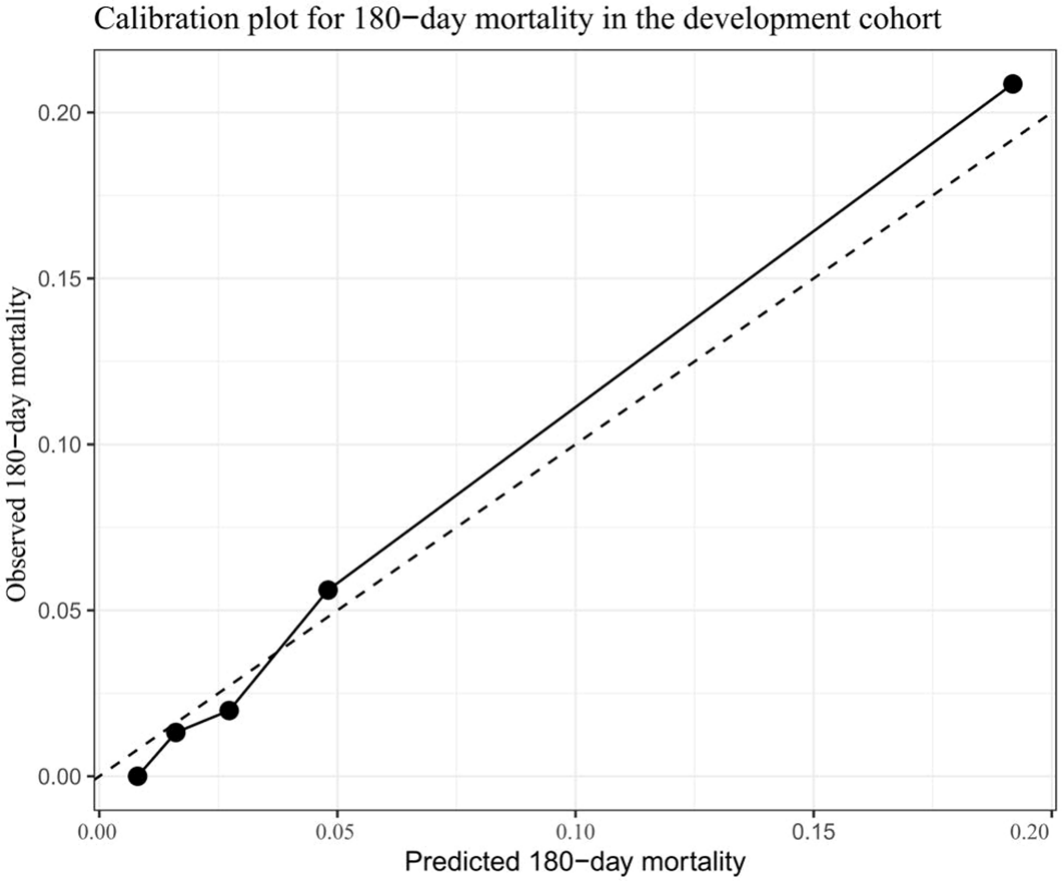
Calibration plot of predicted versus observed 180-day mortality in the development cohort. Calibration plot of predicted versus observed 180-day mortality in the development cohort. Patients were grouped into quintiles according to predicted risk. The dashed line represents the line of identity, and solid points indicate observed mortality within each risk group. Predicted and observed risks were in overall agreement across groups.

In 1,000 bootstrap resamples, the optimism-corrected C-index was 0.838 and the optimism-corrected calibration slope was 0.947, indicating limited optimism and good internal stability. Grouped calibration analysis showed good agreement between predicted and observed mortality across risk strata (**Figure 5**; **Supplementary Table 5**). Overall, these findings suggest that the model had good internal validity with minimal overfitting.

### 3.6 Comparison with GRACE and external validation

We formally compared the final model with GRACE in the development DM-AMI cohort. The final eight-variable model achieved an AUC of 0.857, which was close to that of GRACE (AUC, 0.875), and the difference was not statistically significant (delta AUC, -0.018; DeLong P = 0.292). When the final model linear predictor was added to GRACE, the combined model achieved an AUC of 0.889 and modestly but statistically improved discrimination compared with GRACE alone (delta AUC, 0.014; DeLong P = 0.031). Decision curve analysis also suggested a modest net-benefit advantage for the combined model across most examined threshold probabilities. In the MIMIC-IV validation cohort after endpoint reconstruction, 563 of 2,155 patients died within 180 days. The final model maintained external discriminative performance (AUC, 0.744), with a Brier score of 0.203 (**Table 5**).

**Table 5:** External validation of the 180-day mortality endpoint.

Metric	Estimate
N	2,155
Reconstructed 180-day deaths	563
Corrected 180-day mortality	26.1%
AUC at 180 days	0.744 (0.720-0.768)
Brier score at 180 days	0.203
Calibration slope	0.527
Calibration intercept	-3.998
Observed/expected event ratio	0.652

External Validation of the 180-Day Mortality Endpoint. Data are presented as estimates with 95% confidence intervals (CI), where applicable. The MIMIC-IV endpoint was reconstructed to include post-discharge deaths within 180 days. AUC, area under the receiver operating characteristic curve; O/E, observed-to-expected event ratio.

**Figure 6:**
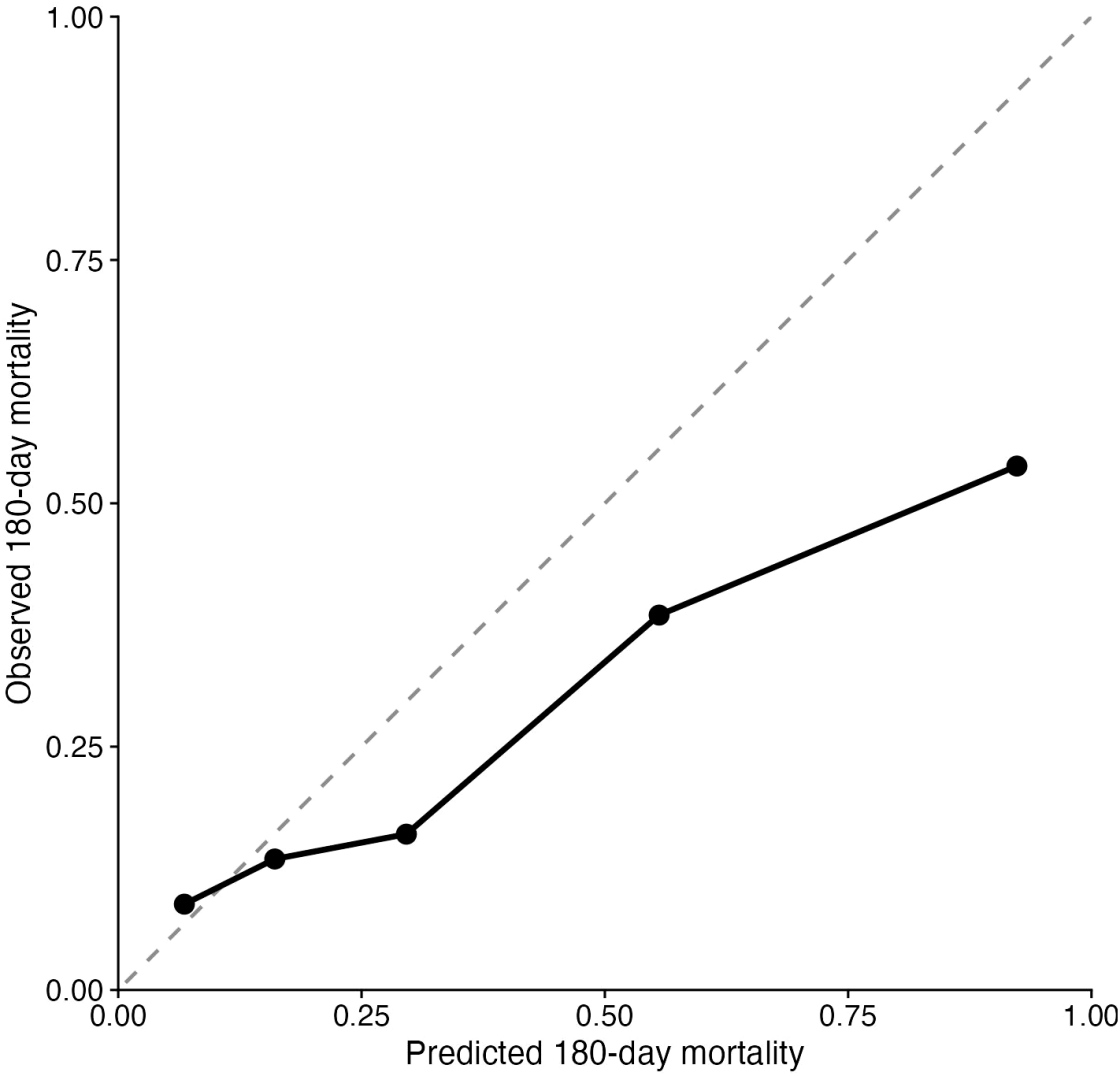
Calibration of the final model after reconstruction of the 180-day mortality endpoint in MIMIC-IV. Calibration plot of predicted versus observed 180-day mortality for the final model in the external validation after endpoint reconstruction cohort. The MIMIC-IV endpoint was reconstructed to include post-discharge deaths within 180 days. Patients were grouped into quintiles according to predicted risk. The dashed line represents the line of identity, and solid points indicate observed mortality within each risk group.

Calibration assessment in MIMIC-IV showed a setting-dependent shift in absolute risk estimates (**Figure 6**). The calibration slope was 0.527, and the mean predicted risk was 40.1% compared with an observed 180-day mortality rate of 26.1%, corresponding to an O/E ratio of 0.652. Overestimation was most apparent in higher predicted-risk groups. Compared with the derivation cohort, the ICU-based MIMIC-IV validation cohort was older and had lower systolic blood pressure and hemoglobin but higher glucose, BUN, creatinine, RDW, and WBC levels, indicating substantial case-mix heterogeneity that likely contributed to the calibration difference.

## 4 Discussion

This dual-cohort study characterized admission risk heterogeneity among patients with DM-AMI and developed a concise prediction model for 180-day all-cause mortality. The final model retained eight routinely available admission variables representing age, hemodynamic status, metabolic stress, renal function, hematologic reserve, and systemic inflammation. In the development cohort, the model showed good discrimination, calibration, and clear risk stratification performance. In the MIMIC-IV cohort, it maintained discriminative ability despite being applied to a substantially different ICU-based population. These findings suggest that routinely collected admission variables can capture clinically meaningful heterogeneity within DM-AMI that is not fully reflected by the diagnostic label of diabetes alone, even in the early phase of hospitalization.

Diabetes is a well-established adverse prognostic factor after AMI, but the presence of diabetes alone does not adequately describe the diversity of early clinical risk within this population (19). Prior studies have shown that patients with diabetes and AMI often carry a heavier burden of comorbidity, metabolic derangement, renal dysfunction, and inflammatory activation than patients without diabetes (20–22). Our findings extend this concept by showing that, even within the DM-AMI subgroup, admission profiles separate patients into markedly different 180-day risk strata. This supports a shift from treating DM-AMI as a uniform high-risk category toward identifying clinically relevant within-group heterogeneity.

The retained predictors are clinically plausible and align with established mechanisms of adverse outcomes after AMI. Age, heart rate, and systolic blood pressure reflect baseline vulnerability and hemodynamic stress at presentation. Glucose may capture acute metabolic stress rather than chronic glycemic exposure alone, which is consistent with the lack of independent prognostic contribution from HbA1c in the final model (23, 24). BUN reflects renal dysfunction and impaired perfusion, whereas hemoglobin, RDW, and WBC provide information on oxygen-carrying capacity, hematologic dysregulation, and systemic inflammation (25–28). Thus, the model does not simply reproduce a single disease-severity score, but integrates several biological domains relevant to DM-AMI prognosis (29, 30).

The comparison with GRACE should be interpreted in this context. GRACE remains a standard and extensively validated tool for ACS risk assessment, and our results do not suggest that it should be replaced (31, 32). Instead, the final model showed discrimination close to GRACE, while the combined GRACE plus model approach improved discrimination and decision-curve net benefit. This indicates that routinely available metabolic, renal, hematologic, and inflammatory variables may contribute information that is complementary to conventional ACS risk scoring, particularly in the diabetes-associated AMI subgroup (33).

Restricted cubic spline analyses suggested nonlinear associations for BUN and hemoglobin. Although a nonlinear sensitivity model improved apparent discrimination and model fit, this improvement came at the cost of additional parameters and a lower events-per-parameter ratio (27, 34–38). Given the limited number of deaths in the derivation cohort, the primary model retained linear terms to preserve parsimony, interpretability, and transportability. The nonlinear findings should be viewed as hypothesis-generating and may inform future model refinement in larger datasets.

External validation in MIMIC-IV provides an important stress test for transportability. The validation cohort was ICU-based and differed from the derivation cohort in illness severity, healthcare setting, and case mix. Under these conditions, preservation of discrimination suggests that the model retained its ability to rank patients by relative risk, whereas the calibration shift indicates that absolute predicted probabilities require adaptation when the model is applied to populations with different baseline mortality and care settings. Consistent reporting of prediction models requires discrimination, calibration, and clinical usefulness to be interpreted as related but distinct aspects of model performance. Therefore, local validation and recalibration should be considered before applying the model for absolute risk prediction in populations substantially different from the derivation cohort.

## 5 Limitations

This study has several limitations. First, the derivation cohort was retrospective and came from a single center, which may affect generalizability. Second, although the final model had an events-per-variable ratio of 11.25 and showed limited optimism in bootstrap validation, only 90 deaths occurred in the derivation cohort; prospective evaluation in larger cohorts would further strengthen the evidence. Third, the MIMIC-IV validation cohort was ICU-based and differed substantially from the derivation cohort in case mix, disease severity, and healthcare setting. This difference provides a stringent external validation scenario but also explains why recalibration is needed when the model is used for absolute risk prediction in other settings. Fourth, several potentially important predictors were unavailable, inconsistently defined, or not harmonizable across cohorts, including troponin, BNP or NT-proBNP, infarct size, coronary anatomy, revascularization timing, medication use, diabetes duration, glycemic control history, and long-term treatment adherence. Fifth, temporal changes in AMI management during the study period may have influenced prognosis, and temporal validation was not performed. Prospective validation is needed to determine whether integrating the model with established clinical assessment improves risk communication, monitoring intensity, or patient outcomes.

## 6 Conclusion

Patients with DM-AMI exhibit substantial risk heterogeneity already at hospital admission. A parsimonious eight-variable model incorporating age, heart rate, systolic blood pressure, glucose, blood urea nitrogen, hemoglobin, red cell distribution width, and white blood cell count captured this heterogeneity using routinely available information. The model achieved discrimination comparable to GRACE in the development cohort, improved prognostic assessment when combined with GRACE, and maintained external discriminative ability in the ICU-based MIMIC-IV cohort. These findings support its role as a practical admission-based framework for refining DM-AMI risk stratification, with local recalibration recommended when absolute risk estimates are used for clinical decision-making.

## Data Availability

The datasets supporting the conclusions of this article are included within the article and its Supplementary Material. Data from the MIMIC-IV database are publicly available at https://doi.org/10.13026/hxp0-hg59. Access requires registration and successful completion of the CITI program. The clinical cohort dataset analyzed during the current study contains sensitive patient information and is therefore not publicly available; de-identified data may be made available from the corresponding author on reasonable request and subject to institutional approval and applicable data-use agreements.
