# Supplementary material for: Development and external validation of an admission-based model for 180-day mortality in diabetic acute myocardial infarction
Source: Front Endocrinol (Lausanne). 2026 Jul 2;17:1891663. doi: 10.3389/fendo.2026.1891663 (PMC13372586; doi:10.3389/fendo.2026.1891663)
Supplement: Supplementary file 1 [file DataSheet1.docx]

**Supplementary Materials**

**Tables:**

**Supplementary Table S1. Univariable Cox Regression Analysis of Admission Variables for 180-Day Mortality in Patients with Diabetic AMI**

| Variable | HR (95% CI) | *P value* |
| --- | --- | --- |
| Age | 1.056 (1.036–1.077) | <0.001 |
| Sex | 0.595 (0.390–0.907) | 0.016 |
| Heart rate | 1.029 (1.020–1.038) | <0.001 |
| SBP | 0.969 (0.958–0.980) | <0.001 |
| STEMI | 1.561 (0.999–2.440) | 0.050 |
| Glucose | 1.115 (1.085–1.145) | <0.001 |
| HbA1c | 1.059 (0.938–1.195) | 0.354 |
| Creatinine | 1.002 (1.001–1.003) | <0.001 |
| BUN | 1.105 (1.085–1.125) | <0.001 |
| Hemoglobin | 0.974 (0.967–0.981) | <0.001 |
| RDW | 1.414 (1.242–1.610) | <0.001 |
| WBC | 1.177 (1.126–1.231) | <0.001 |
| Platelet count | 1.003 (1.000–1.006) | 0.021 |
| Albumin | 0.865 (0.825–0.906) | <0.001 |
| hs-CRP | 1.034 (1.021–1.046) | <0.001 |
| IL-6 | 1.004 (1.003–1.005) | <0.001 |
| D-dimer | 1.200 (1.126–1.278) | <0.001 |
| Killip class 2 | 5.181 (2.508–10.703) | <0.001 |
| Killip class 3 | 16.215 (7.717–34.071) | <0.001 |
| Killip class 4 | 68.257 (33.326–139.802) | <0.001 |
| GRACE score | 1.038 (1.033–1.044) | <0.001 |
| LVEF | 0.952 (0.938–0.967) | <0.001 |

Supplementary Table S1. Univariable Cox Regression Analysis of Admission Variables for 180-Day Mortality in Patients with Diabetic AMI. Data are presented as hazard ratios (HR) with 95% confidence intervals (CI). HRs were derived from univariable Cox proportional hazards regression analysis. Killip class I was used as the reference category. AMI, acute myocardial infarction; SBP, systolic blood pressure; HbA1c, glycated hemoglobin; BUN, blood urea nitrogen; RDW, red cell distribution width; WBC, white blood cell count; hs-CRP, high-sensitivity C-reactive protein; IL-6, interleukin 6; GRACE, Global Registry of Acute Coronary Events; LVEF, left ventricular ejection fraction; STEMI, ST-segment elevation myocardial infarction.

**Supplementary Table S2. Multivariable Cox Regression Analysis of Candidate Admission Variables for 180-Day Mortality Before Derivation of the Final Model**

| Variable | HR (95% CI) | *P value* |
| --- | --- | --- |
| Age | 1.041 (1.020–1.063) | <0.001 |
| Sex | 1.038 (0.650–1.656) | 0.876 |
| Heart rate | 1.017 (1.007–1.027) | <0.001 |
| SBP | 0.979 (0.969–0.990) | <0.001 |
| STEMI | 1.113 (0.692–1.790) | 0.660 |
| Glucose | 1.064 (1.025–1.105) | 0.001 |
| Creatinine | 1.000 (0.998–1.002) | 0.867 |
| BUN | 1.054 (1.008–1.101) | 0.020 |
| Hemoglobin | 0.984 (0.972–0.995) | 0.006 |
| RDW | 1.262 (1.044–1.525) | 0.016 |
| WBC | 1.093 (1.033–1.156) | 0.002 |
| Albumin | 0.964 (0.913–1.018) | 0.191 |

Supplementary Table S2. Multivariable Cox Regression Analysis of Candidate Admission Variables for 180-Day Mortality Before Derivation of the Final Model. Data are presented as hazard ratios (HR) with 95% confidence intervals (CI). HRs were derived from multivariable Cox proportional hazards regression analysis. AMI, acute myocardial infarction; SBP, systolic blood pressure; BUN, blood urea nitrogen; RDW, red cell distribution width; WBC, white blood cell count; STEMI, ST-segment elevation myocardial infarction.

**Supplementary Table S3. Tests for Nonlinearity of Continuous Variables Using Restricted Cubic Spline Analysis**

| Variable | Overall P value | *Nonlinear P value* |
| --- | --- | --- |
| Glucose | <0.001 | 0.220 |
| BUN | <0.001 | <0.001 |
| Hemoglobin | <0.001 | <0.001 |
| RDW | <0.001 | 0.130 |

Supplementary Table S3. Tests for Nonlinearity of Continuous Variables Using Restricted Cubic Spline Analysis. Overall P values represent the association between each variable and 180-day mortality, while nonlinear P values assess deviation from linearity. RCS models were fitted using Cox proportional hazards regression. BUN, blood urea nitrogen; RDW, red cell distribution width.

**Supplementary Table S4. Bootstrap Validation of the Multivariable Model in the Development Cohort**

| Metric | Apparent | Optimism-corrected |
| --- | --- | --- |
| C-index | 0.848 | 0.838 |
| Calibration slope | 1.000 | 0.947 |
| Bootstrap repetitions | 1,000 | 1,000 |

Supplementary Table S4. Bootstrap Validation of the Final Eight-Variable Model in the Development Cohort. Model performance was internally validated using 1,000 bootstrap resamples. Apparent and optimism-corrected estimates are presented.

**Supplementary Table S5. Grouped Calibration of the Multivariable Model in the Development Cohort**

| Calibration group | n | Predicted mortality | Observed mortality |
| --- | --- | --- | --- |
| 1 | 303 | 0.008 | 0.000 |
| 2 | 303 | 0.016 | 0.013 |
| 3 | 303 | 0.027 | 0.020 |
| 4 | 303 | 0.048 | 0.056 |
| 5 | 302 | 0.192 | 0.209 |

Supplementary Table S5. Grouped Calibration of the Multivariable Model in the Development Cohort. Patients were grouped according to predicted 180-day mortality risk. Predicted mortality represents the mean predicted risk within each group, and observed mortality represents the corresponding observed event rate.

**Supplementary Table S6. Grouped Calibration of the Multivariable Model in the External Validation Cohort**

| Calibration group | n | Predicted mortality | Observed mortality |
| --- | --- | --- | --- |
| 1 | 431 | 0.068 | 0.088 |
| 2 | 431 | 0.161 | 0.135 |
| 3 | 431 | 0.296 | 0.160 |
| 4 | 431 | 0.556 | 0.385 |
| 5 | 431 | 0.923 | 0.538 |

Supplementary Table S6. Grouped Calibration of the Final Eight-Variable Model After Reconstruction of the 180-Day Mortality Endpoint in MIMIC-IV. Patients were grouped into quintiles according to predicted 180-day mortality risk. The MIMIC-IV endpoint was reconstructed to include post-discharge deaths within 180 days.

Supplementary Table S7. Comparison of the Final Model, GRACE Score, and Combined Model in the Development Cohort

| Model | N | Events | C-index (95% CI) | 180-day AUC (95% CI) | AUC difference vs GRACE | P value |
| --- | --- | --- | --- | --- | --- | --- |
| Final 8-variable model | 1,514 | 90 | 0.848 (0.809-0.887) | 0.857 (0.817-0.897) | -0.018 | 0.292 |
| GRACE score | 1,514 | 90 | 0.867 (0.831-0.902) | 0.875 (0.838-0.912) | Reference | Reference |
| GRACE + final 8-variable model | 1,514 | 90 | 0.880 (0.846-0.914) | 0.889 (0.854-0.924) | 0.014 | 0.031 |

Supplementary Table S7. Comparison of the Final Model, GRACE Score, and Combined Model in the Development Cohort. AUC differences and P values were calculated using DeLong tests, with GRACE as the reference model. CI, confidence interval; AUC, area under the receiver operating characteristic curve.

**Supplementary Table S8. Baseline Characteristics of Patients in the External Validation Cohort**

| Characteristic | Value |
| --- | --- |
| N | 2155 |
| Age, years | 71.6 (63.5–79.3) |
| Sex, n (%) |  |
| Female | 766 (35.5) |
| Male | 1389 (64.5) |
| Heart rate, bpm | 82.9 (73.3–91.7) |
| SBP, mmHg | 114.0 (106.3–124.2) |
| STEMI, n (%) |  |
| No | 1050 (48.7) |
| Yes | 1105 (51.3) |
| PCI, n (%) |  |
| No | 1221 (56.7) |
| Yes | 934 (43.3) |
| AF, n (%) |  |
| No | 1356 (62.9) |
| Yes | 799 (37.1) |
| CKD, n (%) |  |
| No | 895 (41.5) |
| Yes | 1260 (58.5) |
| Glucose, mmol/L | 10.6 (7.7–15.3) |
| BUN, mmol/L | 9.64 (6.07–16.06) |
| Hemoglobin, g/L | 103 (91–117) |
| RDW, % | 14.8 (13.8–16.3) |
| WBC, ×10⁹/L | 12.2 (9.2–16.2) |
| Creatinine, µmol/L | 114.9 (79.6–203.3) |
| SOFA score | 5 (3–8) |
| APSIII score | 45 (34–60) |
| SAPSII score | 39 (31–48) |
| OASIS score | 33 (27–39) |
| CCI | 7 (6–9) |
| 180-day mortality, n (%) |  |
| No | 1592 (73.9) |
| Yes | 563 (26.1) |

Supplementary Table S8. Baseline Characteristics of Patients in the External Validation Cohort. Data are presented as median (interquartile range, IQR) for continuous variables or n (%) for categorical variables. SBP, systolic blood pressure; PCI, percutaneous coronary intervention; AF, atrial fibrillation; CKD, chronic kidney disease; BUN, blood urea nitrogen; RDW, red cell distribution width; WBC, white blood cell count; SOFA, Sequential Organ Failure Assessment; APSIII, Acute Physiology Score III; SAPSII, Simplified Acute Physiology Score II; OASIS, Oxford Acute Severity of Illness Score; CCI, Charlson Comorbidity Index.

**Supplementary Table S9. Extended Baseline Characteristics of Patients With AMI According to Diabetes Status**

| Characteristic | Total (n=4167) | non-DM (n=2653) | DM (n=1514) | *P*-value |
| --- | --- | --- | --- | --- |
| Age, years | 63.00(55.00,71.00) | 62.00(54.00,70.00) | 64.00(57.00,72.00) | <0.0001 |
| Sex, n (%) |  |  |  | <0.0001 |
| Female | 952(22.85) | 517(19.49) | 435(28.73) |  |
| Male | 3215(77.15) | 2136(80.51) | 1079(71.27) |  |
| Height, cm | 170.00(163.00,173.00) | 170.00(164.73,173.00) | 169.00(160.00,173.00) | <0.0001 |
| Weight, kg | 72.00(65.00,80.00) | 72.00(65.00,80.00) | 71.73(65.00,80.00) | 0.18 |
| HR, bpm | 76.00(68.00,86.00) | 75.00(66.00,84.00) | 78.00(70.00,88.00) | <0.0001 |
| SBP, mmHg | 129.00(116.00,144.00) | 129.00(116.00,144.00) | 130.00(117.00,144.00) | 0.05 |
| DBP, mmHg | 75.00(66.00,85.00) | 75.00(67.00,85.00) | 74.00(66.00,83.00) | <0.0001 |
| GLU, mmol/L | 6.23(5.26,8.16) | 5.71(5.05,6.56) | 8.28(6.24,11.61) | <0.0001 |
| HbA1c, % | 6.10(5.60,7.00) | 5.76(5.50,6.20) | 7.38(6.30,8.68) | <0.0001 |
| Cr, µmol/L | 73.00(63.00,89.70) | 73.00(63.00,87.00) | 73.00(62.00,89.70) | 0.13 |
| BUN, mmol/L | 5.80(4.62,7.04) | 5.57(4.47,6.69) | 6.35(4.91,7.68) | <0.0001 |
| Hb, g/L | 138.00(128.00,149.00) | 140.00(130.00,150.00) | 137.13(124.00,145.00) | <0.0001 |
| WBC, ×10⁹/L | 9.17(7.42,11.06) | 9.24(7.44,11.23) | 9.10(7.37,10.72) | 0.01 |
| RDW | 12.90(12.40,13.20) | 12.90(12.50,13.30) | 12.90(12.40,13.20) | 0.11 |
| PLT, ×10⁹/L | 223.00(185.00,273.34) | 225.00(187.00,273.34) | 221.00(181.00,273.34) | 0.12 |
| ALB, g/L | 37.15(34.88,39.63) | 37.35(34.88,39.92) | 36.63(34.56,39.12) | <0.0001 |
| PA, mg/L | 228.09(193.38,263.41) | 234.00(201.00,269.00) | 220.41(182.00,253.00) | <0.0001 |
| hs-CRP, mg/L | 14.76(3.45,28.56) | 12.45(3.15,26.77) | 19.15(4.21,31.28) | <0.0001 |
| IL-6, pg/mL | 23.26(12.08,40.63) | 22.52(11.75,39.47) | 24.53(12.73,42.48) | <0.01 |
| D-Dimer, µg/mL | 0.35(0.23,0.75) | 0.32(0.21,0.66) | 0.41(0.25,0.79) | <0.0001 |
| CK-MB, U/L | 84.00(20.00,267.00) | 94.00(21.60,272.83) | 63.75(19.00,262.38) | <0.0001 |
| CK, U/L | 185.00(83.00,747.50) | 218.00(89.00,874.00) | 144.32(75.00,542.00) | <0.0001 |
| LDH, U/L | 317.85(205.00,507.71) | 317.85(207.00,531.00) | 305.50(202.00,454.00) | <0.01 |
| ALT, U/L | 29.00(18.00,43.00) | 29.05(18.00,46.00) | 27.00(17.00,38.00) | <0.0001 |
| AST, U/L | 45.27(27.00,125.00) | 51.00(28.00,141.00) | 45.27(25.00,96.00) | <0.0001 |
| TBL, µmol/L | 13.01(10.03,16.79) | 13.01(10.37,17.25) | 13.01(9.37,16.01) | <0.0001 |
| UA, µmol/L | 356.00(296.00,423.73) | 361.00(302.00,423.73) | 344.00(285.00,423.73) | <0.0001 |
| Hcy, µmol/L | 15.30(12.10,21.60) | 15.60(12.30,21.92) | 14.90(11.80,20.40) | <0.0001 |
| HDL-C, mmol/L | 0.97(0.82,1.16) | 0.99(0.84,1.17) | 0.94(0.82,1.13) | <0.0001 |
| LDL-C, mmol/L | 2.51(2.13,3.14) | 2.60(2.17,3.19) | 2.37(2.03,3.04) | <0.0001 |
| TG, mmol/L | 1.45(1.13,2.12) | 1.41(1.09,2.06) | 1.53(1.22,2.25) | <0.0001 |
| TC, mmol/L | 4.12(3.64,4.87) | 4.21(3.64,4.92) | 3.97(3.58,4.78) | <0.0001 |
| Na, mmol/L | 141.00(139.00,142.21) | 141.00(139.00,142.31) | 140.20(138.00,142.00) | <0.0001 |
| K, mmol/L | 4.09(3.82,4.31) | 4.08(3.82,4.30) | 4.11(3.83,4.34) | 0.01 |
| GRACE Score | 150.00(128.00,175.00) | 147.00(125.00,172.00) | 155.00(134.00,180.00) | <0.0001 |
| CRUSADE Score | 22.00(13.00,36.00) | 19.00(10.00,30.00) | 30.00(19.00,43.00) | <0.0001 |
| LVEF, % | 59.00(51.00,63.00) | 59.00(52.00,63.00) | 58.00(50.00,62.23) | <0.0001 |
| STEMI, n (%) |  |  |  | <0.0001 |
| No | 1482(35.57) | 863(32.53) | 619(40.89) |  |
| Yes | 2685(64.43) | 1790(67.47) | 895(59.11) |  |
| AF, n (%) |  |  |  | 0.79 |
| No | 4013(96.30) | 2557(96.38) | 1456(96.17) |  |
| Yes | 154( 3.70) | 96( 3.62) | 58( 3.83) |  |
| COPD, n (%) |  |  |  | 0.78 |
| No | 4148(99.54) | 2642(99.59) | 1506(99.47) |  |
| Yes | 19( 0.46) | 11( 0.41) | 8( 0.53) |  |
| Smoking, n (%) |  |  |  | <0.0001 |
| No | 2532(60.76) | 1545(58.24) | 987(65.19) |  |
| Yes | 1635(39.24) | 1108(41.76) | 527(34.81) |  |
| CKD, n (%) |  |  |  | <0.0001 |
| No | 3809(91.41) | 2482(93.55) | 1327(87.65) |  |
| Yes | 358( 8.59) | 171( 6.45) | 187(12.35) |  |
| PVD, n (%) |  |  |  | 0.45 |
| No | 4148(99.54) | 2643(99.62) | 1505(99.41) |  |
| Yes | 19( 0.46) | 10( 0.38) | 9( 0.59) |  |
| Killip class, n (%) |  |  |  | <0.0001 |
| 1 | 2658(63.79) | 1794(67.62) | 864(57.07) |  |
| 2 | 1092(26.21) | 631(23.78) | 461(30.45) |  |
| 3 | 312( 7.49) | 179( 6.75) | 133( 8.78) |  |
| 4 | 105( 2.52) | 49( 1.85) | 56( 3.70) |  |
| HTN, n (%) |  |  |  | <0.0001 |
| 0 | 1663(39.91) | 1211(45.65) | 452(29.85) |  |
| 1 | 287( 6.89) | 178( 6.71) | 109( 7.20) |  |
| 2 | 739(17.73) | 426(16.06) | 313(20.67) |  |
| 3 | 1478(35.47) | 838(31.59) | 640(42.27) |  |
| PCI, n (%) |  |  |  | <0.001 |
| No | 1528(36.67) | 918(34.60) | 610(40.29) |  |
| Yes | 2639(63.33) | 1735(65.40) | 904(59.71) |  |
| Culprit vessel, n (%) |  |  |  | 0.49 |
| LAD | 2218(53.23) | 1405(52.96) | 813(53.70) |  |
| LCX | 640(15.36) | 408(15.38) | 232(15.32) |  |
| RCA | 1289(30.93) | 824(31.06) | 465(30.71) |  |
| LM | 20(0.48) | 16(0.60) | 4(0.26) |  |

Supplementary Table S9. Extended Baseline Characteristics of Patients With AMI According to Diabetes Status. This table provides the full baseline variable list from the original Table 1; the main Table 1 was shortened to improve readability.

**Figures:**

Supplementary Figure S1. Decision curve analysis comparing GRACE, the final model, and the combined model


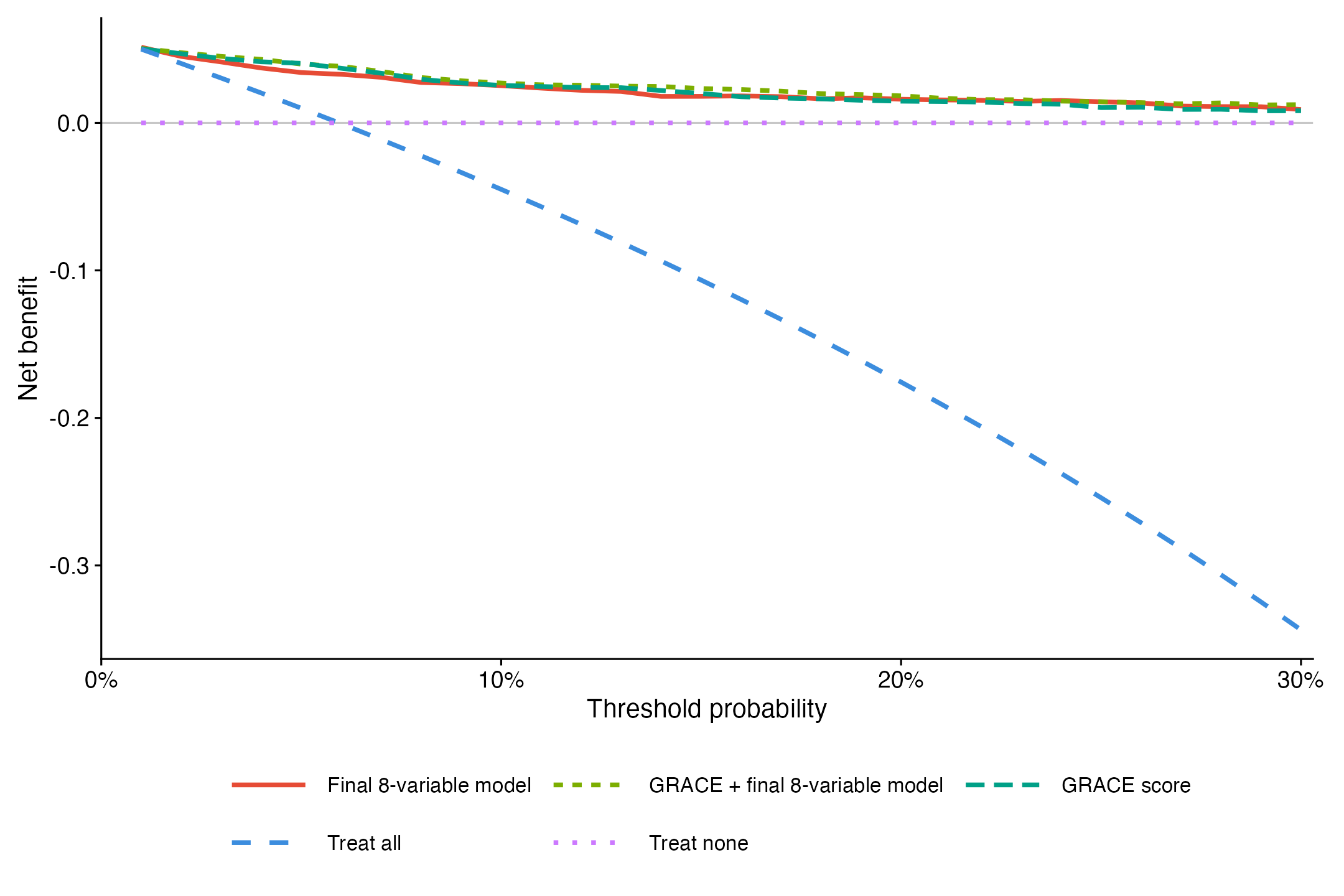


Supplementary Figure S1. Decision curve analysis comparing GRACE, the final eight-variable model, and the combined GRACE plus final model in the development cohort. Net benefit was evaluated across clinically relevant threshold probabilities.
